# Dysregulated DNA Methylation in *Abca4^-/-^* Retinal Pigment Epithelium: Insights into Early Stage of Stargardt Disease

**DOI:** 10.3390/ijms262110742

**Published:** 2025-11-05

**Authors:** Arpita Dave, Anela Tosevska, Marco Morselli, Emily Tom, Matteo Pellegrini, Dorota Skowronska-Krawczyk, Roxana A. Radu

**Affiliations:** 1 UCLA Jules Stein Eye Institute and Department of Ophthalmology, David Geffen School of Medicine, University of California, Los Angeles, CA 90095, USA; arpita2269@g.ucla.edu; 2 Department of Molecular, Cell, and Developmental Biology, University of California, Los Angeles, CA 90095, USA; anela.tosevska@meduniwien.ac.at (A.T.); marco.murslegn@gmail.com (M.M.); matteop@mcdb.ucla.edu (M.P.); 3 Department of Internal Medicine 3, Division of Rheumatology, Medical University of Vienna, 1090 Vienna, Austria; 4 Robert M. Brunson Center for Translational Vision Research, Department of Physiology and Biophysics, Department of Ophthalmology and Visual Sciences, School of Medicine, University of California, Irvine, CA 92697, USA; etom2@hs.uci.edu

**Keywords:** recessive Stargardt disease, retinal pigment epithelium, DNA methylation, methyl-CpG-binding protein 2, reduced representation bisulfite sequencing

## Abstract

Stargardt disease (STGD1), the most common inherited juvenile macular degeneration, is caused by biallelic mutations in the *ABCA4* gene. Currently, there is no approved treatment. In this study, we investigated early-stage epigenomic changes in the retinal pigment epithelium (RPE) of *Abca4^-/-^* mice, a well-established model of STGD1. Reduced representation bisulfite sequencing (RRBS) revealed hypermethylation of gene regions associated with disease-related pathways, implicating methyl-CpG-binding protein 2 (MeCP2) and RE1-silencing transcription factor (REST) as potential regulators. Notably, DNA methylation of a subset of genes preceded their transcriptional change and disease phenotypes in *Abca4^-/-^* RPE. Together with the detected age-dependent increase in MeCP2 levels in *Abca4^-/-^* RPE, these findings suggest that early DNA methylation changes may contribute to RPE dysfunction and eventual cell loss in STGD1.

## 1. Introduction

Loss of retinal pigment epithelial cell (RPE) structural and functional integrity causes death of photoreceptors, leading to macular dystrophy in recessive Stargardt disease (STGD1) [1,2,3,4]. Mutations in the ATP-binding cassette transporter (*ABCA4*) gene are responsible for STGD1, a juvenile blinding disease [5]. Currently, there are *no* efficacious treatments for STGD1 [6,7]. While ABCA4 is well known for its role in photoreceptors, it is also expressed and functions in the membranes of RPE cells [8,9,10,11,12,13,14]. Studies in RPE cells derived from patients with *ABCA4* mutations and *Abca4* knock-out mice display disease-associated phenotypes, such as increasing accumulation of vitamin A dimers (bisretinoids), complement system dysregulation, and endolysosomal and mitochondrial dysfunctions [15,16,17,18,19], all of which are mediated by differential gene expressions.

DNA methylation, an epigenetic modification that involves adding methyl groups to cytosine residues of DNA in CpG (cytosine-phosphate-guanine) sites, is a dynamic process for the regulation of gene expression. The majority of CpGs are found within CpG islands, the DNA regions with GC content greater than 50% and are associated with 70% of annotated gene promoters [20]. DNA methylation was first identified as the DNA modification involved in gene repression [21]; however, more recent studies demonstrate that DNA methylation acts as context-dependent regulation, functioning both as a gene repressor and an activator based on its location in the gene [22,23]. The regulation of gene transcription by DNA methylation is mediated by proteins that recognize CpG duplexes involving (1) DNA methyl region-binding proteins, (2) DNA methylases or methyltransferases, and (3) DNA demethylases [24].

Methyl-CpG-binding 2 (MeCP2), a protein that binds methylated CpGs [25], has been extensively studied in the central nervous system (CNS) due to its mutations linked to Rett syndrome, a neurodevelopmental disorder [26]. In CNS cells, MeCP2 deficiency and overexpression have been reported to induce a spectrum of dysfunctions through the role of MeCP2 in transcription activation, repression, chromatin remodeling, and RNA splicing [27,28,29,30,31,32,33]. Embryologically, the CNS, retina, and RPE all originate from the neuroectoderm [34], sharing common pathways for their development. In the eye, MeCP2 expression in photoreceptors begins during the postnatal period (P0–P16), whereas in the RPE, it is detectable as early as the embryonic stage (E12) and persists throughout life [35]. On a functional level, MeCP2 binds to the Transforming Growth Factor-beta (TGF-β) gene promoter in RPE cells [36,37] and regulates epithelial–mesenchymal transition (EMT) [38,39], a key pathogenic mechanism linked to age-related macular degeneration (AMD) [40]. Additionally, reduced MeCP2 levels impair RPE ciliogenesis [41], a process essential for RPE development and stress response. Disruption of ciliogenesis was linked to retinal degeneration [42]. Despite these findings, the potential role of MeCP2 in both the neural retina and RPE in the context of disease remains unknown.

To test the hypothesis that early DNA methylation changes contribute to RPE dysfunction in STGD1 through MeCP2-mediated mechanisms, we performed reduced representation bisulfite sequencing (RRBS), transcriptomic analyses, and MeCP2 protein level assessment on RPE/eyecups from *Abca4^-/-^* and wild-type mice. Our results suggest that DNA methylation, together with the co-transcriptional regulators MeCP2 and RE1-silencing transcription factor (REST), contributes to the progressive dysregulation of the transcriptomic makeup of RPE cells and age-dependent molecular modifications in *Abca4^-/-^* mice. These findings provide insight into how altered DNA methylation patterns in the initial stages may contribute to disease progression in STGD1, informing us on the underlying mechanistic pathways of cell death and guiding the development of novel therapeutic strategies.

## 2. Results

### 2.1. RRBS Identified Altered DNA Methylation in RPE of 1-Month-Old Abca4^-/-^ Versus Wild-Type 129/Sv Mice

**Figure 1:**
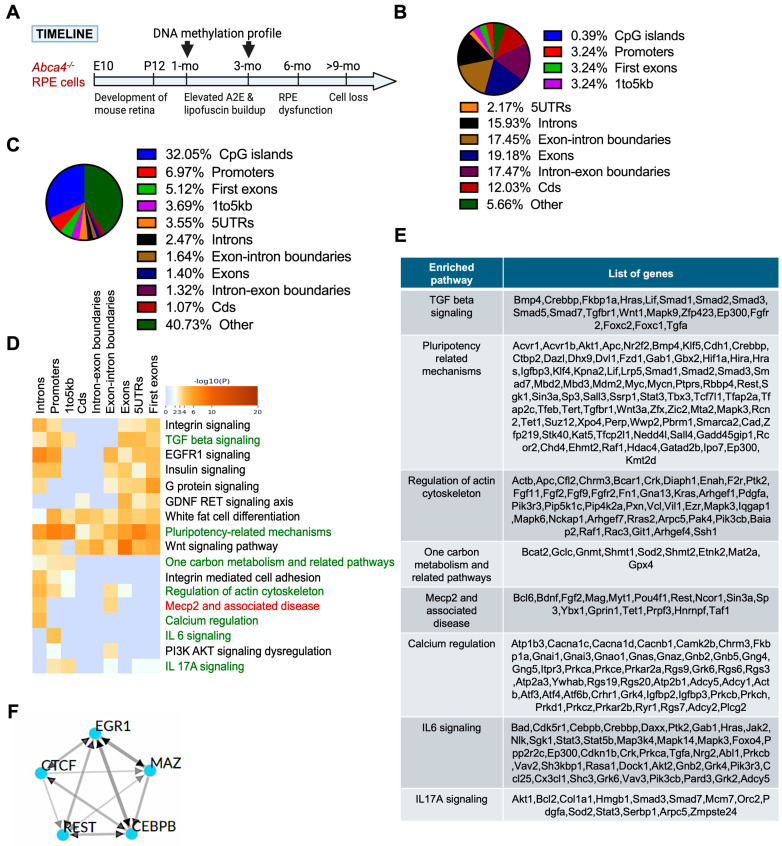
RRBS analysis of the RPE from pigmented (129/Sv) 1-month-old *Abca4^-/-^* mice. (**A**) Timeline of retinal development and STGD1 disease phenotypes in *Abca4^-/-^* mice. Retina development of mice occurs during embryonic (E) day 10 to postnatal (P) day 12 [60]. Within the first month, bisretinoids (A2E) and lipofuscin-autofluorescence buildup are initiated in the RPE of mice lacking ABCA4, resulting in significantly higher levels of A2E at 3 months, which leads to dysfunction and death of the RPE from an age greater than 6 months. RRBS was performed at 1 month and 3 months to capture this initial stage of disease development. (**B**) Percentage distribution of CpG sites (CpGs) covered across the genome regions in all samples: 1-month-old and 3-month-old *Abca4^-/-^* and wild-type mice (both 129/Sv and BALB/c strains) by the RRBS analysis. (**C**) Percentage distribution of differentially methylated CpGs (DMCs) located at diverse genomic regions in pigmented 1-month-old *Abca4^-/-^* versus wild-type RPE. (**D**) Gene ontology and pathway enrichment analysis of hypermethylated CpGs-associated genes in pigmented 1-month-old *Abca4^-/-^* versus wild-type RPE. (**E**) Hypermethylated gene list of key regulatory pathways affected, from (**D**). (**F**) Transcription factors associated with hypermethylated CpGs in promoters in the RPE of pigmented 1-month-old *Abca4^-/-^* versus wild-type mice (3 mice/genotype/strain).

*Abca4^-/-^* mice recapitulate key features of the STGD1 phenotype, exhibiting detectable levels of bisretinoid–autofluorescence in the RPE as early as 1 month of age [19,43]. By 6 months, RPE dysfunction is evident, and by 9 to 12 months, loss of RPE and photoreceptor cells is reported [12,43,44]. To investigate early DNA methylation changes, we analyzed differentially methylated CpGs (DMCs) in RPE tissue from age-matched 1- and 3-month-old wild-type and *Abca4^-/-^* mice (Figure 1A). Genome-wide profiling of all CpG sites (CpGs) revealed the following distribution: 0.39% in CpG islands, 3.24% in promoters, 3.24% in first exons, 3.24% in proximal regulatory region (1–5 kb upstream of transcriptional start site), 2.17% in 5′ untranslated regions (UTR), 15.93% in introns, 19.18% in exons, 17.47% in intron–exon, 17.45% at exon–intron boundaries, 12.03% in coding sequence, and 5.66% in other regions (including lncRNA, intergenic, CpG shores, CpG shelves, and enhancers) captured by RRBS (Figure 1B), consistent with distribution described by RRBS analysis [45].

Differential analysis of RPE from 1-month-old *Abca4^-/-^* compared to the age- and background-matched wildtype mice demonstrated that DMCs were predominantly enriched in proximal regulatory elements such as CpG islands (32%), promoters (6.97%), first exons (5.12%), and 1 to 5 kb gene regions (3.69%) (Figure 1C). Enrichment analysis of DMCs revealed that hypermethylated CpGs in the RPE of *Abca4^-/-^* mice, in both 129/Sv and BALB/c strains, were associated with genes involved in key pathways related to RPE physiology. This finding prompted us to focus on hypermethylated CpGs in 1-month and 3-month-old mice. Specifically, genes associated with hypermethylated CpGs were significantly enriched in pathways related to RPE function, cellular signaling, and neurodegenerative processes. Markedly, enriched pathways included MeCP2 and its related disorders (*p*-value = 8.59 × 10^−5^), TGF-β signaling (*p*-value = 2.64 × 10^−5^), pluripotency regulation (*p*-value = 4.10 × 10^−12^), actin cytoskeleton dynamics (*p*-value = 5.11 × 10^−7^), calcium signaling (*p*-value = 2.07 × 10^−5^), one-carbon metabolism (*p*-value = 7.42 × 10^−4^), IL-6 signaling (*p*-value = 5.08 × 10^−5^), and IL-17A signaling (*p*-value = 4.01 × 10^−4^) in 1 month *Abca4^-/-^* versus wild-type RPE (Figure 1D,E), all previously linked to neurodegenerative diseases and dysfunction of RPE in AMD [46,47,48,49,50,51].

### 2.2. Transcription Factors Associated with Hypermethylated DMC-Promoters in RPE of Abca4^-/-^ Versus Wild-Type 129/Sv Mice

To identify potential upstream regulators affected by an increased DNA methylation in *Abca4^-/-^* RPE, we performed ChEA3 transcription factor binding site enrichment analysis [52] using promoter sequences corresponding to significantly hypermethylated CpG site-associated genes in 1-month-old *Abca4^-/-^* animals compared to age- and background-matched wild-type ones. This analysis revealed significant enrichment of binding sites for EGR1, CTCF, REST, CEBPB, and MAZ (Figure 1F). These transcription regulators are known to function as site-specific regulators (EGR1 and MAZ) [53,54], transcription co-activators (CEBPB) [55], and chromatin organizers (CTCF and REST) [56,57]. Notably, REST has been previously shown to interact with MeCP2 and has been implicated in epigenetic regulation underlying neurodegeneration [58,59].

### 2.3. RNA-Seq Analysis Identified Transcriptional Changes in RPE from 1-Month-Old Abca4^-/-^ Compared to Age-Matched 129/Sv Wild-Type Mice

**Figure 2:**
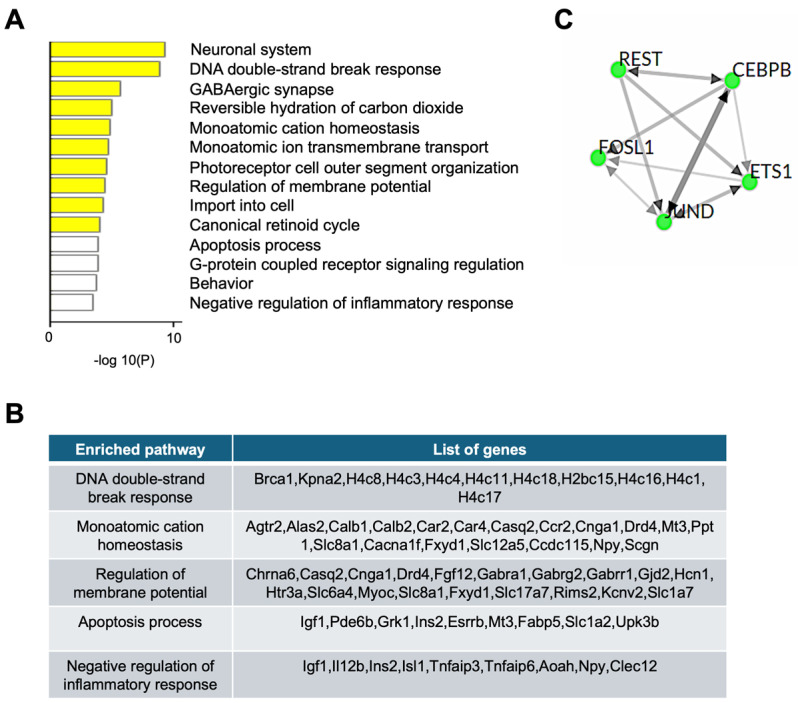
RNA-seq analysis of the RPE from pigmented (129/Sv) 1-month-old *Abca4^-/-^* mice. (**A**) Gene ontology and pathway enrichment analysis of downregulated genes extracted from RNA-seq dataset (accession number: GSE63772). Yellow and white bars indicate varying levels of statistical significance. (**B**) Downregulated genes list of key regulatory pathways affected, from (**A**). (**C**) Transcription factors associated with downregulated genes in *Abca4^-/-^* compared to wild-type mice (3 biological replicates).

To assess the extent to which the hypermethylation of proximal regulatory regions correlates with transcriptional downregulation in the RPE of 1-month-old *Abca4^-/-^* mice, we analyzed a publicly available RNA-seq dataset (GSE63772). Differentially expressed genes (DEGs) were identified using a threshold of fold change > 1 and adjusted *p*-value < 0.05. Gene ontology analysis revealed significant downregulation of genes involved in DNA repair (*p*-value = 3.6 × 10^−6^), monoatomic cation homeostasis (*p*-value = 4.3 × 10^−5^), regulation of membrane potential (*p*-value = 3.2 × 10^−5^), apoptotic processes (*p*-value = 1.5 × 10^−4^), and negative regulation of inflammatory response (*p*-value = 3.7 × 10^−4^) (Figure 2A,B). ChEA3 analysis of regulatory elements for downregulated genes identified FOSL1, JUND, ETS1, CEBPB, and REST as the top five transcription factors binding motifs associated with these changes (Figure 2C). Remarkably, analysis of hypermethylated regions at the same stage of disease revealed a REST binding site (Figure 1F), a key co-factor of MeCP2, underscoring the critical role of MeCP2-REST complex in driving early transcriptional reprogramming in STGD1 RPE.

### 2.4. DNA Methylation-Enriched Pathways in the RPE Cells of 3-Month-Old Abca4^-/-^ Mice

**Figure 3:**
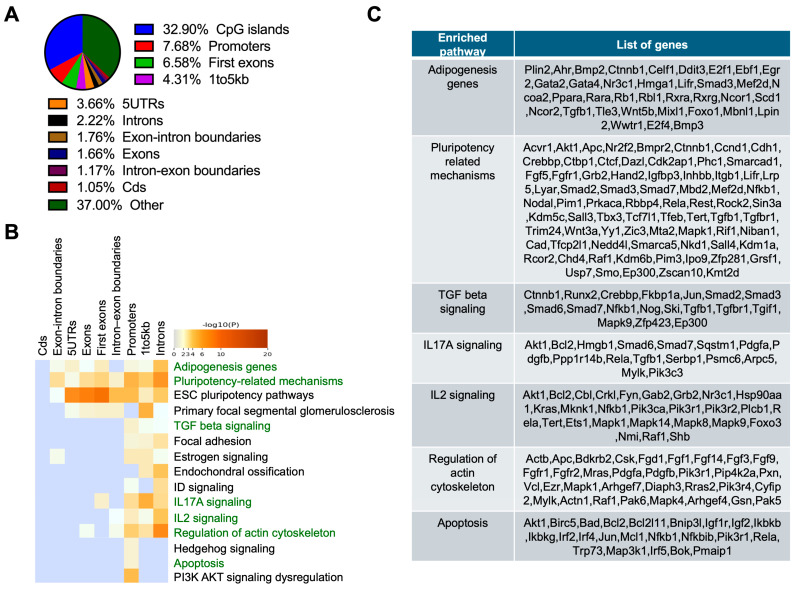
RRBS analysis of the RPE from pigmented (129/Sv) 3-month-old *Abca4^-/-^* mice. (**A**) Percentage distribution of differentially methylated CpGs (DMCs) located at diverse genomic regions in pigmented 3-month-old *Abca4^-/-^* versus wild-type RPE. (**B**) Gene ontology and pathway enrichment analysis of hypermethylated CpG site-associated genes in pigmented 3-month-old *Abca4^-/-^* versus wild-type RPE. (**C**) Hypermethylated gene list of key regulatory pathways affected, from (**B**) (3 mice/genotype).

**Figure 4:**
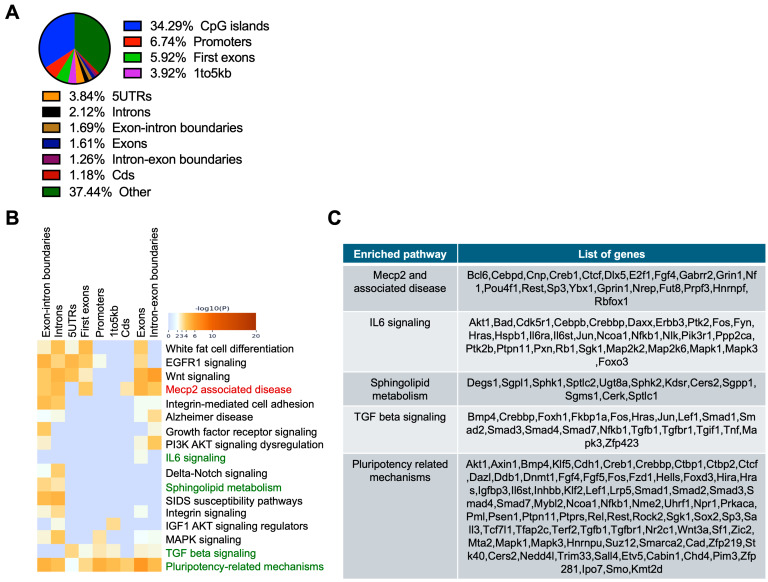
RRBS analysis of the RPE from albino (BALB/c) 3-month-old *Abca4^-/-^* mice. (**A**) Percentage distribution of differentially methylated CpGs (DMCs) located at diverse genomic regions in albino 3-month-old *Abca4^-/-^* versus wild-type RPE. (**B**) Gene ontology and pathway enrichment analysis of hypermethylated CpG-associated genes in albino 3-month-old *Abca4^-/-^* versus wild-type RPE. (**C**) Hypermethylated gene list in key regulatory pathways affected, from (**B**) (3 mice/genotype).

To understand the progression of changes in DNA methylation in disease, RRBS analysis was performed on RPE tissue from 3-month-old *Abca4^-/-^* mice on 129/Sv (pigmented) (Figure 3) and BALB/c (albino) (Figure 4) backgrounds, along with respective wild-type controls. In both models, the highest enrichment of DMCs was detected in proximal regulatory regions. DMCs were primarily located in CpG islands (32.90%), followed by promoters (7.68%), first exons (6.58%), and regions 1–5 kb upstream of genes (4.31%) in *Abca4^-/-^* pigmented mice (Figure 3A). Enrichment analysis of hypermethylated CpGs-associated genes revealed pathways related to lipid synthesis (adipogenesis; *p*-value = 1.36 × 10^−6^), key signaling pathway TGF-β (*p*-value = 1.31 × 10^−5^), IL-17A (*p*-value = 6.73 × 10^−6^), IL-2 (*p*-value = 5.27 × 10^−8^), actin cytoskeleton regulation (*p* = 6.36 × 10^−8^), maintenance of pluripotency (*p*-value = 2.44 × 10^−11^), and apoptosis (*p*-value = 2.88 × 10^−5^) (Figure 3B,C).

In the RPE of 3-month-old *Abca4^-/-^* mice on BALB/c (albino) background, 34.29% of DMCs were located within CpG islands, followed by 6.74% in promoter regions, 5.92% in first exons, and 3.92% in 1–5 kb upstream regions (Figure 4A). Hypermethylated CpG-located genes were significantly enriched in pathways associated with MeCP2-related disorders (*p*-value = 2.1 × 10^−8^), IL-6 signaling (*p*-value = 8.3 × 10^−7^), TGF-β signaling (*p*-value = 8.7 × 10^−7^), and pluripotency regulation (*p*-value = 3.8 × 10^−13^) (Figure 4B,C), partially aligning with findings of 1-month and 3-month *Abca4^-/-^* RPE on 129/Sv backgrounds (Figure 1D and Figure 3B). Also, sphingolipid metabolism, which is essential for RPE cell homeostasis and has been implicated in AMD pathogenesis [61,62,63], exhibits significant hypermethylation (*p*-value = 9.3 × 10^−6^) in *Abca4^-/-^* RPE.

### 2.5. Age-Dependent Increase of MeCP2 Protein in Abca4^-/-^ Mice RPE and in Human RPE

**Figure 5:**
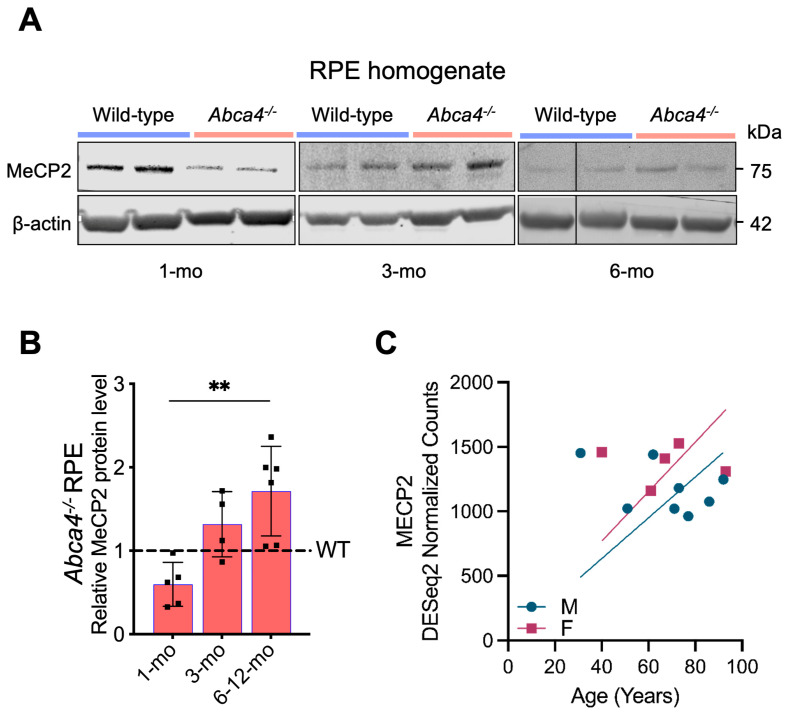
Immunoblotting analysis of MeCP2 protein in RPE of pigmented (129/Sv) *Abca4^-/-^* mice and human MeCP2 RNA-seq data. (**A**) Representative immunoblots for MeCP2 and *β*-actin as internal control in 1-, 3-, and 6-month (mo)-old *Abca4^-/-^* and wild-type mice RPE/eyecup (30 μg total protein/lane). (**B**) Level of MeCP2 protein normalized to *β*-actin is plotted relative to wild-type (WT) levels, with 6 mo and 12 mo sample data combined. The experiment was repeated twice (5–6 mice/genotype). Data are represented as mean ± SD; adjusted ** *p* < 0.01; two-way ANOVA with Bonferroni correction. (**C**) MeCP2 gene expression in human (male—M, female—F) RPE with aging, extracted from the GEO database (accession number: GSE159435).

The observed deregulation of MeCP2-related genes prompted us to evaluate MeCP2 expression in RPE. Longitudinal immunoblot analysis revealed a striking ~2.5-fold increase in MeCP2 protein levels in *Abca4^-/-^* mouse RPE from 1 to ~6 months of age, compared to age-matched 129/Sv wild-type controls (Figure 5A,B). After 6 months, MeCP2 levels remained persistently elevated at ~2-fold above wild-type levels (Figure 5A,B). These results demonstrate a sustained, age-dependent dysregulation of MeCP2 in *Abca4^-/-^* RPE, implicating this epigenetic regulator as a potential driver of progressive RPE dysfunction. Supporting its translational relevance, analysis of publicly available data (GSE159435) [64] showed that MeCP2 transcript levels also increase with age in human RPE tissue (Figure 5C), suggesting a conserved age-associated role for MeCP2 in RPE homeostasis.

## 3. Discussion

This study serves as a *proof-of-concept* that epigenetic dysregulation, mediated by altered DNA methylation and MeCP2-REST signaling, contributes to early RPE pathology and highlights the need to understand how epigenetic factors, alongside *ABCA4* genetic mutations, influence disease progression and severity in Stargardt patients. STGD1 is a highly heterogeneous macular dystrophy in terms of age of onset, disease progression, and clinical presentations [65,66]. The genetic and phenotypic variability poses challenges for diagnosis and treatment, and cell-specific pathophysiological processes responsible for ABCA4-mediated maculopathies are still understudied.

DNA methylation changes are increasingly recognized as key epigenetic modifications involved in the pathogenesis of AMD, a related disease manifesting with RPE degeneration [67,68,69,70]. Multiple studies have shown altered DNA methylation patterns in the RPE and peripheral blood of AMD patients, including hypomethylation of genes such as IL17RC, which leads to increased expression and is associated with AMD progression [71,72]. Genome-wide methylation profiling reveals numerous differentially methylated regions in AMD samples, affecting genes that regulate oxidative stress responses, inflammation, and metabolic pathways [73,74,75]. This epigenetic dysregulation can modulate gene expression crucial for RPE cell function and survival, contributing to retinal degeneration, highlighting the dynamic interplay between genetics, environment, and epigenetics in initiating the pathology and disease progression [73,76,77,78,79,80]. To date, epigenetic regulation has not been investigated in materials from STGD1 animal models or patient-derived RPE cells.

Here, we examined DNA methylation dynamics in the RPE cells of *Abca4^-/-^* mice and identified early, disease-relevant epigenetic changes that precede overt structural or functional decline. Although RPE integrity is preserved at 1 month of age, we observed differential DNA methylation in CpG islands, promoters, and first exons of key genes, implicating early epigenetic priming in disease initiation. These changes intensified by 3 months in two STGD1 mouse models (pigmented and albino *Abca4^-/-^* mice), paralleling increased A2E-bisretinoid levels and autofluorescence [19,43,81,82], suggesting a progressive, methylation-mediated shift in RPE homeostasis. The pronounced changes in DNA methylation, predominantly at the proximal regulatory regions in *Abca4^-/-^* mice RPE, suggest a mechanism related to the deregulation of gene transcription activation rather than mRNA splicing.

While ABCA4 mutations are mainly studied in the context of STGD1, a subset of patients with AMD, geographic atrophy, and cone-rod dystrophies are also linked to ABCA4 variants [83,84,85,86,87]. Many of the identified hypermethylated genes in *Abca4^-/-^* mice RPE are involved in pathways previously linked to aging and AMD, including cytokine signaling, cytoskeleton regulation, and cell metabolism [75,88]. For instance, hypermethylation of actin cytoskeleton-associated genes may impair RPE phagocytic capacity [89], while IL-6/IL-17A and TGF-β signaling pathways could exacerbate inflammation, fibrosis, and EMT, hallmarks of AMD, a disease clinically related to STGD1. Specifically, methylation changes in genes related to pluripotency and developmental regulation indicate possible dedifferentiation of *Abca4^-/-^* RPE cells and a higher probability of migration. These findings position DNA methylation as a potential driver of RPE dysfunction in STGD1, with relevance to other related retinal diseases.

Transcriptomic analysis further supports this interpretation. In 1-month-old *Abca4^-/-^* RPE, gene expression changes were already evident in pathways regulating double-strand DNA repair, membrane potential, and inflammatory responses, indicating early, broader molecular dysregulation beyond bisretinoid toxicity caused by ABCA4 deficiency. While other studies have proposed a function for MeCP2 in maintaining the non-neuronal identity of the RPE [36,37,38,70], notably, our methylome-transcriptome analysis revealed enrichment for dysregulation of REST-mediated repression mechanisms, suggesting a role of MeCP2 in the RPE homeostasis. Interestingly, while dysregulation of inflammation and inflammation-related pathways could be detected on both transcriptional and epigenetic levels, several pathways differentially affected by hypermethylation in 1-month RPE of *Abca4^-/-^* were not deregulated at the transcriptional level at the same age. Precisely, hypermethylation was detected on genes involved in cell–cell adhesion and cytoskeleton remodeling, despite the absence of corresponding transcriptional or phenotypical changes. Notably, in STGD1, RPE cells undergo morphological and functional alterations driven by bisretinoid accumulation, oxidative stress, and complement dysregulation, which together disrupt cell adhesion and cytoskeleton integrity [12,19,90]. Emerging evidence from our study suggests that hypermethylation of genes governing these pathogenic pathways may represent an early step of disease-related changes in RPE, highlighting a potential epigenetic layer of gene regulation that amplifies the pathology.

We also found that MeCP2 protein levels progressively increased with age in *Abca4^-/-^* RPE, suggesting a role of MeCP2 in stabilizing hypermethylated chromatin and repressing transcription. Given MeCP2’s ability to act as a context-dependent transcriptional regulator, it may exert wide-ranging effects on RPE gene networks. However, the overwhelming enrichment of DNA methylation changes in proximal regulatory regions suggests its key role in transcriptional repression. In addition, the concurrent downregulation of genes with REST, a co-repressor often recruited by the MeCP2 binding site at their promoters, suggests the MeCP2-REST complex as a key component regulating transcriptional changes in the tissue. REST, known to silence neuronal genes in non-neuronal tissue [91,92,93], therefore plays a similar role in RPE [94]. However, it is also known to act as a transcription activator by recruiting TET3 hydroxylase and NSD3, a chromatin remodeler, to its promoters. Specifically, recruitment of TET3 to the REST-binding site causes conversion of methyl cytosine to 5-hydroxymethylcytosine, resulting in transcriptional activation [93]. In addition, REST-dependent recruitment of NSD3, a histone methyltransferase, promotes methylation of H3K36 residue, associated with active transcription [93]. With dynamic changes in DNA methylation patterns and increasing levels of MeCP2 in the tissue, REST’s role as a transcriptional repressor may shift to new promoter regions, including those normally associated with active gene expression in healthy tissue. Therefore, this potential of MeCP2 and REST in establishing the transcriptional profile of diseased tissue adds a novel epigenetic dimension to the pathology of STGD1 and positions the MeCP2–REST complex as a candidate driver of age-related RPE functional decline.

While our integrative approach offers valuable insights, several limitations must be acknowledged. First, our observations are primarily correlative, and the causal impact of the methylation changes on RPE dysfunction remains to be experimentally validated. Whether low-level bisretinoid accumulation (1-month) directly alters epigenetic regulators, or whether epigenetic dysregulation occurs independently in the *Abca4^-/-^* RPE, is still unknown. Second, although MeCP2 upregulation has been observed in *Abca4^-/-^* mice RPE, and its genomic targets and co-factors have been mapped in human iPSC RPE [70], the MeCP2–REST regulatory network in murine and human STGD1 RPE models remains to be characterized. This underscores the need for MeCP2 chromatin immunoprecipitation sequencing (ChIP-seq) studies in conjunction with age-stratified transcriptomic analysis. Third, the findings are currently limited to the RPE of the *Abca4^-/-^* STGD1 mouse model. Validation in human STGD1 RPE cells is essential to establish translational relevance. Lastly, while our DNA methylation study focuses on early stages (1–3 months), longitudinal profiling is necessary to understand how epigenetic regulation evolves throughout phenotype buildup to the stage that evidences RPE cell loss. Given the overlap in gene pathways between STGD1 and AMD [12,95,96,97,98,99,100], these findings also support the utility of the STGD1 models in studying age-related retinal degeneration more broadly.

In summary, our results reveal that DNA methylation changes and MeCP2-REST-mediated transcriptional repression occur before detectable RPE dysfunction in the STGD1 mouse model, suggesting they may play a role in disease progression. These findings not only provide a new understanding of epigenetic mechanisms in STGD1 but also highlight common molecular pathways with AMD. In conclusion, our findings reveal that DNA methylation changes in disease-related pathways contribute to the molecular mechanisms underlying RPE damage in null ABCA4-associated STGD1 and AMD. However, how specific *ABCA4* mutations influence DNA methylation patterns or are themselves modulated by epigenetic alterations remains to be determined using patient-derived RPE models. Future studies dissecting the interplay between epigenetic regulation and ABCA4 mutation type may elucidate the basis of phenotypic heterogeneity observed among patients. These findings support RPE cell replacement therapy for STGD1 and AMD, given that epigenetic factors change in diseased RPE.

## 4. Materials and Methods

### 4.1. Mice

One-month- and three-month-old age-, sex-, and background-matched wild-type (129/Sv and BALB/c) and Abca4-null (*Abca4^-/-^*) mice on the same wild-type backgrounds (129/Sv and BALB/c) were used in this study. These *Abca4^-/-^* lines were generated by Dr. Gabriel Travis and Dr. Roxana A. Radu, as previously reported in [16,101]. Mice housed in normal cyclic 12 h light/12 h dark conditions were fed ad libitum with a standard rodent diet and were genotyped to confirm as negative for the *Abca4* alleles. All experiments followed the ARVO Statement for the Use of Animals in Ophthalmic and Vision Research and UCLA IACUC guidelines.

### 4.2. Collection of RPE/Eyecup

Mice were euthanized by cervical dislocation, and eyes were harvested for obtaining RPE/eyecup separated from neural retina. In 1× phosphate buffer saline (PBS, pH 7.4), the anterior segment of the eye was removed, followed by the separation of the neural retina from the eyecup [10]. RPE/Eyecups were stored in a −80 °C freezer until further processing.

### 4.3. DNA Extraction and Quantification

DNA was extracted from mice, one RPE/eyecup per sample, using the Dneasy Blood & Tissue Kit (Qiagen, Hilden, Germany, Cat# 69506). DNA was eluted in 50 μL AE Buffer. The concentration was measured with a Qubit instrument (Life Technologies, Carlsbad, CA, USA), and 100 ng of genomic DNA was treated with RNAse enzyme at 37 °C for 30 min to remove contaminating RNA.

### 4.4. Library Preparation and Reduced Representation Bisulfite Sequencing (RRBS)

RRBS libraries were prepared by a Zymo-Seq RRBS Library kit (Zymo research, Irvine, CA, USA, Cat# D5460). The libraries were subjected to size selection using magnetic AMPure XP beads (Beckman Coulter Life Sciences, Indianapolis, IN, USA) to enrich DNA fragments of the desired size. The quality control of the final libraries (size-selected and barcoded) was performed using an Agilent TapStation 4200 (D1000, D5000; Agilent Technologies, Santa Clara, CA, USA). Pooled libraries were run on a NovaSeq 6000 as 100 bp single-end reads (Illumina, San Diego, CA, USA).

### 4.5. Data Analysis

Reads were aligned to the mm10 mouse reference genome (GCF_000001635.26) using Bsbolt [102], followed by methylation calling using default parameters. Unsupervised and differential methylation analyses were conducted with RnBeads2 [103]. Briefly, sites were filtered based on coverage of 10 or more across 80% of the samples. Here, 653489 sites met the criteria. Methylation values were calculated as a value between 0 and 1, where 0 is not methylated and 1 is fully methylated. One sample was flagged as an outlier and removed from downstream analysis. Differential methylation analysis was performed using hierarchical linear models from the limma package and fitted using an empirical Bayes model [104] as implemented in the RnBeads2 package. Sites were considered differentially methylated if they reached an FDR-corrected significance level below 0.05 and a difference in the methylation value between the two groups of at least 10%. Differentially methylated sites were then annotated using the annotatr package in R [105] and ChipSeeker [106]. Percentage distribution of CpGs and DMCs on genomic regions was derived from the summarize_annotations() function from the annotatr package, and the results were represented as percentages of the total number of CpG sites. Gene ontology and enrichment analysis were performed using clusterProfiler [107] and Metascape (v3.5.20250701) [108] with the mouse reference genome as a background.

### 4.6. RNA-Sequencing Data Set Enrichment Analysis

The RNA-seq dataset list of the differentially expressed genes was acquired from NCBI database (BioProject accession: PRJNA269047 [109]. Upregulated and downregulated expressed genes in *Abca4^-/-^* versus wild-type were sorted based on the log2 (fold change) and significance level *p* < 0.05. Gene pathway enrichment analysis was performed using Metascape software (v3.5.20250701) [108] with the mouse reference genome as a background.

### 4.7. Immunoblotting

RPE/Eyecups from 1-, 3-, 6-, and 12-month-old *Abca4^-/-^* and age–gender-matched 129/Sv wild-type mice were collected in 1× PBS with 1× Halt protease inhibitor cocktail (Thermo Scientific, Waltham, MA, USA, Cat# 78429). Tissues were lysed by sonication for 20 s. Tissue lysates were incubated with 1× Benzonase nuclease (EMD Millipore, Burlington, MA, USA, Cat# 71205-3) for 1 h, followed by 20 min of 0.5% SDS (Sigma-Aldrich, St. Louis, MI, USA, Cat# L3771) with gentle agitation at room temperature. Separation of protein lysates from cell debris was achieved by spinning at 1000× *g* for 5 min at 4 °C. Total protein was estimated using a Micro BCA Protein Assay Kit (Thermo Scientific, Cat# 23235). An amount of 40 μg of total protein was separated on 12% Bis-Tris polyacrylamide gels (Thermo Scientific, Cat# NP0341BOX) by running it in 1× MOPS buffer at 100 V. Proteins were transferred to a PVDF membrane (EMD Millipore, Cat# IPFL00010) in 1× transfer buffer (Thermo Scientific, Cat# NP00061) using semi-dry transfer cells (Bio-rad, Hercules, CA, USA) for 45 min at 20 V. The membrane was blocked with protein-free Blocking Buffer (LI-COR Biosciences, Lincoln, NE, USA, Cat# 927-90010) at RT for 1 h on a rocker and probed with MeCP2 (Cell Signaling Technology, Danvers, MA, USA, Cat# 3456S; or Abcam, Cambridge, UK, Cat# ab2829) (1:1000 dilution) and β-actin (Thermo Scientific, Waltham, MA, USA, Cat# MA5-15452) (1:1000 dilution) primary antibodies in blocking buffer with 0.5% donkey serum overnight on rocker at 4 °C. The membrane was washed with 1× PBS-T (0.1% Tween 20) 3 times for 5 min. LI-COR IRDye-680 or -800 channel secondary antibody (1:10,000 dilution) in blocking buffer with 0.5% donkey serum, incubation for 1 h at RT on rocker, was performed, followed by a washing step 3 times for 5 min on a rocker. Membrane bands were imaged using an Odyssey CLx Infrared Imaging System and LI-COR software (Version 6.0) (LI-COR Biosciences). Band intensities were quantified with Image Studio Lite Ver 5.2. Statistical analysis was performed using GraphPad Prism 9.

### 4.8. Human Aging RPE Data Set Analysis

Bulk RNA-sequencing data from aging human RPE were obtained from the GEO database (accession number: GSE159435) [64]. Raw read counts were normalized using the DESeq2 package. Gene expression levels of MeCP2 were plotted as a function of donor age, and simple linear regression was performed to assess age-associated expression trends.

## Data Availability

The data generated and analyzed during the current study will be available upon request to radu@jsei.ucla.edu (Roxana A. Radu) or arpita2269@g.ucla.edu (Arpita Dave). Sequencing data have been deposited in the ArrayExpress database at EMBL-EBI (https://www.ebi.ac.uk/biostudies/arrayexpress, accessed on 26 October 2025) under the accession number E-MTAB-16049.

## Abbreviations

The following abbreviations are used in this manuscript:

STGD1	Recessive Stargardt Disease
AMD	Age-Related Macular Degeneration
RRBS	Reduced Representation Bisulfite Sequencing
DMC	Differentially Methylated CpGs
